# Use of sugammadex is associated with reduced incidence and severity of postoperative nausea and vomiting in adult patients with obesity undergoing laparoscopic bariatric surgery: a post-hoc analysis

**DOI:** 10.1186/s12871-023-02123-y

**Published:** 2023-05-15

**Authors:** Xiahao Ding, Xiaozhong Zhu, Cuimei Zhao, Dapeng Chen, Yuting Wang, Hui Liang, Bo Gui

**Affiliations:** 1 Department of Anesthesiology and Perioperative Medicine, First Affiliated Hospital with Nanjing Medical University, Nanjing, 210029 China; 2 Department of Anesthesiology, Affiliated Taikang Xianlin Drum Tower Hospital, Medical School of Nanjing University, Nanjing, 210046 China; 3 Department of Anesthesiology, Nanjing Qixia District Hospital, Nanjing, 210046 China; 4 Department of General Surgery, First Affiliated Hospital with Nanjing Medical University, Nanjing, 210029 China

**Keywords:** Obesity, Laparoscopic bariatric surgery, Postoperative nausea and vomiting, Sugammadex, Propensity score matching

## Abstract

**Background:**

Postoperative nausea and vomiting (PONV) is a common but troublesome complication in patients who undergo laparoscopic bariatric surgery (LBS). Whether sugammadex use is related to the persistent decrease in the occurrence of PONV during postoperative inpatient hospitalization, which is critical for the rehabilitation of patients after LBS, remains unknown.

**Methods:**

The study was based on a randomized controlled trial conducted in an accredited bariatric centre. A total of 205 patients who underwent LBS were included in the analysis. Univariate analysis and multivariable logistic regression model were used to identify the significant variables related to PONV. Then propensity score matching and inverse probability of treatment weighting (IPTW) were employed to compare outcomes between the sugammadex and neostigmine groups. The primary outcome was the incidence of PONV within 48 h after LBS. The secondary endpoints included the severity of PONV, time to first flatus, need for rescue antiemetic therapy, and water intake.

**Results:**

The incidence of PONV was 43.4% (89/205) within the first 48 h after LBS. In multivariable analysis, sugammadex use (OR 0.03, 95% CI 0.01–0.09, *P* < 0.001) was an independent protective factor of PONV. After IPTW adjustment, sugammadex use was associated with lower incidence of PONV (OR 0.54, 95% CI 0.48–0.61, *P* < 0.001), postoperative nausea (PON) (OR 0.77, 95% CI 0.67–0.88, *P* < 0.001), and postoperative vomiting (POV) (OR 0.60, 95% CI 0.53–0.68, *P* < 0.001) within postoperative 48 h. The severity of PON as well as the incidence and severity of POV within the first 24 h were also lower in the sugammadex group (all *P* < 0.05). Reduced need for rescue antiemetic therapy within the first 24 h, increased water intake for both periods, and earlier first passage of flatus were observed in the sugammadex group (all *P* < 0.05).

**Conclusions:**

Compared with neostigmine, sugammadex can reduce the incidence and severity of PONV, increase postoperative water intake, and shorten the time to first flatus in bariatric patients during postoperative inpatient hospitalization, which may play a pivotal role in enhanced recovery.

**Trial registration:**

Chinese Clinical Trial Registry (ChiCTR2100052418, http://www.chictr.org.cn/showprojen.aspx?proj=134893, date of registration: October 25, 2021).

**Supplementary Information:**

The online version contains supplementary material available at 10.1186/s12871-023-02123-y.

## Background

Postoperative nausea and vomiting (PONV) is a common complication following general anesthesia and surgery, particularly in patients undergoing laparoscopic bariatric surgery (LBS), with a prevalence of up to 80% [1, 2]. Despite multimodal PONV prophylaxis, 47.4% of patients required antiemetic rescue medication [3]. Untreated PONV after LBS has severe adverse consequences, including water-electrolyte imbalance, anastomotic fistula, patient dissatisfaction, malnutrition, increased treatment costs, and early hospital readmission [4–6]. Therefore, this common but troublesome complication has attracted increasing attention. It is clinically important to develop strategies for the prevention of PONV in patients undergoing LBS.

Sugammadex, a modified γ-cyclodextrin, has been used clinically to reverse neuromuscular blockade (NMB) of steroidal neuromuscular blocking drugs (NMBDs) [7]. The drug provides fast recovery of neuromuscular function and prevents postoperative residual NMB in patients with severe obesity [8]. Accordingly, sugammadex is recommended for patients with body mass index (BMI) ≥ 35 kg/m^2^ as an NMB reversal agent in our center after obtaining their informed consent. Although there are several studies exploring the effects of sugammadex on PONV, most of the available evidence suggests that sugammadex only tends to reduce the occurrence of PONV; however, these results failed to reach statistical significance [9, 10]. In addition, most of the possible explanations for the relationship between sugammadex and reduction in PONV focuses on the emetic effect of neostigmine [11]. Whether sugammadex use is related to the decreased occurrence of PONV during inpatient hospitalization, which is critical for the rehabilitation of patients undergoing bariatric surgery, remains unknown. For these reasons, independent factors associated with PONV in adult patients with obesity undergoing LBS were identified. Furthermore, we designed a propensity score matching (PSM) analysis to compare the incidence and severity of PONV between sugammadex and neostigmine use in these bariatric patients. We hypothesized that the use of sugammadex might reduce the incidence of PONV during postoperative hospital stay.

## Methods

### Study design and data source

**Fig. 1:**
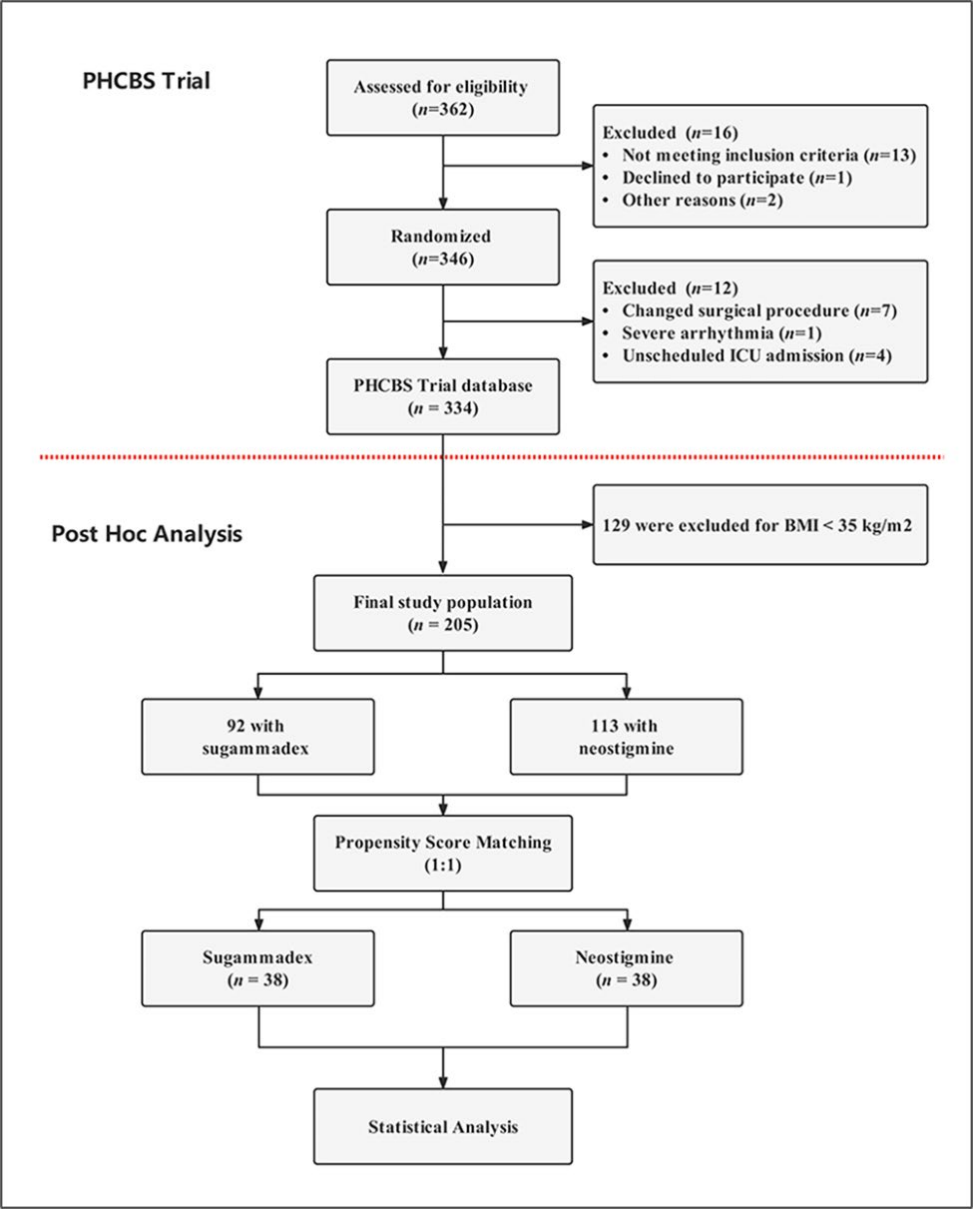
Flow chart of the study. *BMI* body mass index, *ICU* intensive care units

Data were retrieved from the database of our recently completed trial (named PHCBS; IRB #2020-SR-059; registered at http://www.chictr.org.cn/showprojen.aspx?proj=134893, ChiCTR2100052418), which is a prospective, randomized, double-blind, controlled study designed to study the effect of penehyclidine hydrochloride (PHC) on PONV in adult patients scheduled for LBS. Written informed consent was obtained from all subjects participating in the trial. The inclusion criterion for our post hoc analysis was BMI ≥ 35 kg/m^2^. The flow diagram of the study is shown in Fig. 1. A total of 205 patients were included in this post hoc analysis, with 92 and 113 patients in the sugammadex and neostigmine groups, respectively. Baseline demographics, comorbid conditions, simplified Apfel score, surgical information, and intraoperative and postoperative parameters were obtained from the PHCBS trial data.

### Perioperative protocol

The same surgeon performed all procedures including laparoscopic sleeve gastrectomy (LSG), laparoscopic sleeve gastrectomy plus duodenojejunal bypass (LSG-DJB), laparoscopic sleeve gastrectomy plus jejunojejunal bypass (LSG-JJB), and one-anastomosis gastric bypass (OAGB). All patients received standard anesthesia with midazolam, propofol, fentanyl, and rocuronium, or cis-atracurium to facilitate tracheal intubation. Prophylactic dexamethasone (10 mg, intravenously) was administered during induction. Thereafter, the patients were mechanically ventilated with a tidal volume of 6–8 mL/kg (ideal body weight) and a respiratory rate of 12–16 times per min to maintain end-tidal carbon dioxide level of 35–45 mmHg. Anesthesia was maintained with continuous infusion of propofol 100–200 mcg · kg^− 1^ · min^− 1^, remifentanil 0.05–0.15 mcg · kg^− 1^ · min^− 1^, and rocuronium 5–10 mcg · kg^− 1^ · min^− 1^, or cis-atracurium 1–3 mcg · kg^− 1^ · min^− 1^ using lean body weight. NMBDs were selected according to the choice of reversal agent. If NMB was reversed by sugammadex, rocuronium was used for both anesthesia induction and maintenance. If neostigmine was used, cis-atracurium was selected instead. The sevoflurane concentration was adjusted when necessary. Blood pressure and heart rate were maintained at a fluctuation of not more than 20% of the baseline value by adjusting the depth of anesthesia or using vasoactive agents during surgery. All patients received a single intravenous dose of palonosetron (0.25 mg) 30 min before the end of surgery. Continuous infusion of NMBDs was stopped after the deflation of the pneumoperitoneum, while propofol and remifentanil infusions were discontinued at the end of surgery. Afterward, patients were admitted to the post-anesthesia care unit (PACU) with an endotracheal tube for recovery from anesthesia. After the recovery of spontaneous breathing, the residual effects of NMBDs were antagonized by the administration of either sugammadex 200 mg (Sugammadex group) or neostigmine 2 mg and atropine 1 mg (Neostigmine group). Tracheal extubation was performed according to a standardized protocol: total consciousness, stable circulation, respiratory rate < 30 breaths/min, maximal inspiratory pressure < − 20 cmH_2_O, tidal volume > 6 mL/kg, pulse oxygen saturation > 93%, and ability to lift the head for 5 s. Criterion used for patient discharge from the PACU was a modified Aldrete score ≥ 9. After the patients were sent back to the ward, if asked by patients or the episode of vomiting within 24 h was > 5 times, intramuscular injection of metoclopramide 10 mg was administered as an initial rescue antiemetic therapy. If the symptoms persisted one hour after metoclopramide administration, another 10 mg of metoclopramide was administered as a second rescue dose. Notably, not more than 20 mg of metoclopramide was allowed in any 24-h period. A total of 10 mg of dezocine was administered intravenously as rescue analgesia in the ward when the visual analog scale score was ≥ 4. Patients were instructed to drink clear liquids for 24–48 h after LBS, which was gradually increased to 2 L to promote recovery of gastrointestinal function [12].

### Outcome measures

The primary outcome was the incidence of PONV within 48 h after LBS. The secondary endpoints included the severity of PONV, time to first flatus, need for rescue antiemetic therapy, and water intake within 48 h after LBS. PONV was defined as at least one episode of nausea, vomiting, or retching. PONV was evaluated as follows: I = no nausea or vomiting, II = nausea but no vomiting, III = mild to moderate vomiting, and IV = severe and frequent vomiting more than five times within 24 h. The severity of postoperative nausea (PON) was assessed using a numeric rating scale (I = mild, II = moderate, III = severe). The severity of postoperative vomiting (POV) was recorded according to the number of vomiting episodes (I = no vomiting, II = vomiting episodes occurring 1–2 times within 24 h, III = vomiting episodes occurring 3–5 times within 24 h, IV = vomiting episodes occurring more than 5 times within 24 h). The volume of postoperative water intake was measured during the two periods (0–24 h and 24–48 h). The time to first flatus was defined as the time to the first passage of flatus minus the end time of LBS according to the patient’s self-report.

### Statistical analysis

All analyses were performed using R software (version 4.2.0, R Foundation for Statistical Computing, Vienna, Austria) and IBM SPSS Statistics for Windows (version 21.0, SPSS Inc. Chicago, Illinois, USA). Statistical significance was defined by a two-sided *P*-value less than 0.05. Descriptive statistics are presented as mean ± standard deviation (SD) or median with interquartile range (IQR) for continuous variables and as frequencies or proportions for categorical variables. Normal distribution of data was confirmed using the Shapiro–Wilk test. For normally distributed data, an independent Student’s *t*-test was used to assess the significance between the two groups. For data with skewed distribution, the Mann–Whitney *U* test was used. Categorical variables were analyzed using either the chi-square or Fisher’s exact test, as appropriate.

Based on clinical importance, scientific knowledge, previous research, and the actual data that could be collected for the study, we collected data from electronic medical records, which included age, sex, body mass index (BMI), smoking status, history of diabetes mellitus, hypertension, motion sickness and PONV, simplified Apfel score, type of surgery, duration of anesthesia and operation, total fluid input, use of intraoperative opioids, sugammadex, penehyclidine and rescue opioids. Univariate analysis and multiple logistic regression models were used to determine the influence of each variable on PONV. Variables were initially assessed in the univariate analysis, and the variables found significantly (*P* < 0.1) associated with PONV were subsequently included in the multivariable logistic regression model using a stepwise forward selection strategy. Additionally, several known factors according to the prior knowledge and theory were also incorporated into the multivariate logistic regression analysis due to their potentially clinical significance. To improve the robustness of statistical methods, full model and a stepwise backward regression model were also conducted to select independent factors associated with PONV. The Omnibus test within the analysis was used to determine the overall significance of a model.

To minimize possible selection bias between the sugammadex and neostigmine groups, we performed a PSM and inverse probability of treatment weighting (IPTW) method to control for observed confounding and verify the comparative results between the compared groups. First, we predicted the probability of receiving sugammadex for each patient using a logistic regression model by including all potential confounders as covariates. Second, we used the estimated propensity scores as weights for patients who received sugammadex and the inverse of 1 minus the propensity score for patients who received neostigmine, which generated a weighted cohort. Patients who received sugammadex were matched on a 1:1 ratio with patients who received neostigmine using nearest neighbor method within a caliper width of 0.1 of the standard deviation without replacement to yield the well matching results. Afterward, unadjusted differences between the sugammadex and neostigmine groups were characterized using the standardized mean difference. Absolute standardized mean difference (ASD) values above 0.2 were deemed to represent meaningful covariate imbalance. Following IPTW, we used conditional logistic regression analysis to compare clinical outcomes to assess the association between sugammadex and any event of nausea and vomiting, with results expressed as adjusted odds ratio (OR) with 95% confidence intervals (CIs) for categorical data and mean difference (MD) for continuous data. The cumulative incidence of the first passage of flatus was estimated using the Kaplan–Meier method and compared using the log-rank test. We also performed a subgroup analysis to compare the two groups within the first 24 h and 24–48 h after LBS.

Because this is a post-hoc analysis and we have utilized all available patients who met our inclusion and exclusion criteria, we performed a power calculation. We considered it clinically meaningful if logistic regression showed sugammadex had an OR for PONV less than 0.55 compared with receiving neostigmine. Assuming type I error of 0.05 and effective sample sizes of 400 observations after applying IPTW with a 1:1 ratio, we confirmed that the statistical power was 0.82. The sample size justification was performed using Power Analysis & Sample Size software (PASS, version 15.0. NCSS, LLC. Kaysville, Utah, USA).

## Results

Data regarding the demographic and clinical characteristics between patients with and without PONV are summarized in Table 1. The incidence of PONV was 43.4% (89/205) within the first 48 h after LBS. Compared to patients without PONV, patients with PONV were more likely to be women and have a history of motion sickness, less likely to have a history of smoking and diabetes, and presented lower BMI, lower ASA classification and higher Apfel risk score (all *P* < 0.05). Moreover, patients with PONV underwent more LSG, experienced shorter duration of anesthesia and operation, received more opioids intraoperatively, less sugammadex and had less amount of the intraoperative fluid infusion (all *P* < 0.05).

**Table 1:** Demographic and clinical characteristics between patients with and without PONV (*n* = 205)

Characteristics	With PONV (*n* = 89)	Without PONV (*n* = 116)	*P* value
Age (years), mean ± SD	31.0 ± 8.6	32.5 ± 8.2	0.187
Female sex, *n* (%)	63 (70.8)	70 (60.3)	0.003^*a*^
BMI (kg/m^2^), mean ± SD	39.9 ± 4.6	42.7 ± 5.9	< 0.001^*a*^
ASA classification, *n* (%)			
II	43 (48.3)	21 (18.1)	< 0.001^*a*^
III	38 (42.7)	103 (88.8)	
Apfel risk score, *n* (%)			
0	2 (2.2)	9 (7.8)	0.003^*a*^
1	10 (11.2)	30 (25.9)	
2	38 (42.7)	50 (43.1)	
3	25 (28.1)	35 (30.2)	
4	6 (6.7)	0 (0)	
Diabetes mellitus, *n* (%)	18 (20.2)	54 (46.6)	0.003^*a*^
Hypertension, *n* (%)	17 (19.1)	27 (23.3)	> 0.999
Smoking, *n* (%)	10 (11.2)	34 (29.3)	0.017^*a*^
His_PONV, *n* (%)	2 (2.2)	0 (0)	0.155
His_MS, *n* (%)	5 (5.6)	0 (0)	0.009^*a*^
Types of surgery, *n* (%)			
LSG	59 (66.3)	50 (43.1)	< 0.001^*a*^
LSG-JJB	21 (23.6)	62 (53.4)	
LSG-DJB	0 (0)	8 (6.9)	
OAGB	1 (1.1)	4 (3.4)	
Dur_anesthesia (h), mean ± SD	1.5 ± 0.3	1.7 ± 0.4	< 0.001^*a*^
Dur_operation (h), mean ± SD	1.2 ± 0.3	1.4 ± 0.4	< 0.001^*a*^
IOC (mg), mean ± SD	60.3 ± 8.3	56.6 ± 7.1	0.001^*a*^
PHC, *n* (%)	47 (52.8)	83 (71.6)	0.252
Sugammadex, *n* (%)	8 (9.0)	84 (72.4)	< 0.001^*a*^
Total fluid input (ml), mean ± SD	1306.8 ± 323.0	1463.7 ± 374.0	0.016^*a*^
Rescue opioids, *n* (%)	45 (50.6)	75 (64.7)	0.579

Data are presented as mean ± standard deviation (SD), median (IQR) or *n* (%) as appropriate

*Abbreviations: ASA* American Society of Anesthesiologists, *BMI* body mass index, *Dur_anesthesia* duration of the anesthesia, *Dur_operation* duration of the operation, *His_MS* history of motion sickness, *His_PONV* history of PONV, *IOC* intraoperative opioids consumption (as intravenous morphine equivalent), *LSG* laparoscopic sleeve gastrectomy, *LSG-DJB* laparoscopic sleeve gastrectomy plus duodenojejunal bypass, *LSG-JJB* laparoscopic sleeve gastrectomy plus jejunojejunal bypass, *OAGB* one anastomosis gastric bypass, *PHC* penehyclidine hydrochloride administration, *PONV* postoperative nausea and vomiting

^*a*^ Statistically significant (*P* < 0.05)

In the univariate analysis, we identified eleven parameters, including female sex, BMI, ASA classification, diabetes mellitus, smoking, type of surgery, duration of anesthesia and operation, intraoperative opioids consumption, sugammadex use and total fluid input, were correlated to PONV probability. Additionally, several known factors were retained in the model due to their clinical significance, including age, Apfel risk score, history of PONV and motion sickness, and rescue opioids. Multivariable logistic regression analyses using stepwise forward regression model continued to verify that sugammadex use (OR 0.03, 95% CI 0.01–0.09, *P* < 0.001), female sex (OR 2.64, 95% CI 1.16–6.00, *P* = 0.021), and diabetes mellitus (OR 0.24, 95% CI 0.11–0.55, *P* < 0.001) were significantly associated with the occurrence of PONV. In the full model, sugammadex use (OR 0.01, 95% CI 0.01–0.06, *P* < 0.001) and age (OR 0.93, 95% CI 0.87–0.99, *P* = 0.015) were independent factors associated with PONV (Table 2). Besides, sugammadex use was also one of the independent protective factors in the stepwise backward regression model (Supplementary Table 1). The Omnibus test revealed an overall significance of the three different models (all *P* < 0.001).

**Table 2:** Risk factors of PONV using stepwise forward regression and full model (*n* = 205)

Variables	Univariate		Multivariable^*a*^		Full model^*b*^		
	OR (95% CI)	*P* value	OR (95% CI)	*P* value	Coefficients	OR (95% CI)	*P* value
Age	0.98 (0.94–1.01)	0.188			-0.08	0.93 (0.87–0.99)	0.015^*c*^
Female sex	2.70 (1.43–5.08)	0.002^*c*^	2.64 (1.16–6.00)	0.021^*c*^	0.94	2.55 (0.61–10.59)	0.198
BMI	0.90 (0.85–0.96)	0.001^*c*^			-0.01	1.00 (0.88–1.13)	0.979
ASA classification							
II	1	Ref				Ref	
III	5.55 (2.92–10.53)	< 0.001^*c*^			-0.69	0.50 (0.12–2.13)	0.349
Apfel risk score							
0	1	Ref				Ref	
1	1.50 (0.28–8.14)	0.638			0.51	1.66 (0.13–21.42)	0.699
2	3.42 (0.70–16.76)	0.129			0.45	1.58 (0.08–31.69)	0.767
3	3.21 (0.64–16.18)	0.157			0.33	1.38 (0.05–35.52)	0.844
4	N/A	> 0.999			43.65	N/A	> 0.999
Diabetes mellitus	0.37 (0.20–0.70)	0.002^*c*^	0.24 (0.11–0.55)	0.001^*c*^	-0.98	0.38 (0.11–1.30)	0.123
Hypertension	0.95 (0.48–1.89)	0.893			0.58	1.79 (0.59–5.39)	0.301
Smoking	0.37 (0.17–0.81)	0.012^*c*^			-0.57	0.57 (0.11–2.95)	0.501
His_PONV	N/A	> 0.999			-1.06	N/A	> 0.999
His_MS	N/A	> 0.999			-22.21	N/A	> 0.999
Type of surgery							
LSG	1	Ref				Ref	
LSG-JJB	0.29 (0.15–0.54)	< 0.001^*c*^			1.39	4.02 (0.82–19.85)	0.088
LSG-DJB	N/A	> 0.999			-19.28	N/A	> 0.999
OAGB	0.21 (0.02–1.96)	0.171			-18.87	N/A	> 0.999
Dur_anesthesia	0.21 (0.09–0.49)	< 0.001^*c*^			-3.18	0.04 (0.01–5.62)	0.204
Dur_operation	0.23 (0.10–0.54)	0.001^*c*^			-4.88	0.01 (0.01–5.83)	0.150
IOC	0.94 (0.90–0.98)	0.002^*c*^			3.95	51.69 (0.34–7843.66)	0.124
PHC	0.68 (0.38–1.22)	0.196			-0.46	0.63 (0.25–1.57)	0.323
Sugammadex	0.05 (0.02–0.12)	< 0.001^*c*^	0.03 (0.01–0.09)	< 0.001^*c*^	-4.27	0.01 (0.01–0.06)	< 0.001^*c*^
Total fluid input	0.99 (0.98–1.00)	0.003^*c*^			-0.01	1.00 (1.00–1.00)	0.270
Rescue opioids	0.82 (0.46–1.44)	0.484				N/A	

*Abbreviations: ASA* American Society of Anesthesiologists, *BMI* body mass index, *CI* confidence interval, *Dur_anesthesia* duration of the anesthesia, *Dur_operation* duration of the operation, *His_MS* history of motion sickness, *His_PONV* history of PONV, *IOC* intraoperative opioids consumption (as intravenous morphine equivalent), *LSG* laparoscopic sleeve gastrectomy, *LSG-DJB* laparoscopic sleeve gastrectomy plus duodenojejunal bypass, *LSG-JJB* laparoscopic sleeve gastrectomy plus jejunojejunal bypass, *N/A* not applicable, *NMB* cumulative doses of the continuous infusion of neuromuscular blockade (as intravenous cis-atracurium equivalent), *OAGB* one anastomosis gastric bypass, *OR* odds ratio, *PHC* penehyclidine hydrochloride administration, *PONV* postoperative nausea and vomiting

^*a*^ All variables found significantly (*P* < 0.1) associated with PONV by univariate analysis and clinically meaningful factors according to the prior knowledge and theory (Age, Apfel risk score, history of PONV, history of motion sickness, rescue opioids) were inserted into the multivariable logistic regression model using a forward selection strategy. Overall *P* value is less than 0.001 in Omnibus test of model coefficient

^*b*^ Overall *P* value is less than 0.001 in Omnibus test of model coefficient

^*c*^ Statistically significant (*P* < 0.05)

The pre-matching comparison of baseline demographics and perioperative variables between the sugammadex and neostigmine groups were presented in Table 3. Compared to patients in the neostigmine group, patients treated with sugammadex presented higher BMI and greater ASA classification (both *P* < 0.001). Moreover, patients in the sugammadex group underwent more LSG-JJB (*P* < 0.001), experienced longer duration of anesthesia and operation (both *P* < 0.001), received more opioids intraoperatively (*P* = 0.008) and had higher amount of the intraoperative fluid infusion (*P* = 0.001).

**Table 3:** Baseline demographics and perioperative variables before and after matching

Variables	Unmatched					After PSM^*a*^				After IPTW^*b*^
	Sugammadex (*n* = 92)	Neostigmine (*n* = 113)	*P* value	ASD^*c*^		Sugammadex (*n* = 38)	Neostigmine (*n* = 38)	*P* value	ASD^*c*^	ASD^*c*^
*Demographics*										
Age (years)	31.0 ± 7.5	32.7 ± 9.0	0.133	0.214		31.5 ± 9.3	30.6 ± 8.6	0.692	0.109	0.196
Female sex	53 (57.6)	80 (70.8)	0.069	0.278		26 (68.4)	23 (60.5)	0.632	0.160	0.120
BMI (kg/m^2^)	44.7 ± 5.4	39.1 ± 4.4	< 0.001^*d*^	1.137		42.0 ± 4.6	42.2 ± 5.9	0.863	0.039	0.025
*Preoperative variables*										
ASA classification										
II	6 (6.5)	58 (51.3)	< 0.001^*d*^	1.137		6 (15.8)	6 (15.8)	> 0.999	< 0.001	0.051
III	86 (93.5)	55 (48.7)				32 (84.2)	32 (84.2)			
Apfel risk score										
0	5 (5.4)	6 (5.3)	0.822	0.174		3 (7.9)	3 (7.9)	0.944	< 0.001	0.158
1	21 (22.8)	19 (16.8)				7 (18.4)	9 (23.7)		0.125	
2	39 (42.4)	49 (43.4)				16 (42.1)	14 (36.8)		0.107	
3	25 (27.2)	35 (31.0)				12 (31.6)	12 (31.6)		< 0.001	
4	2 (2.2)	4 (3.5)				0 (0)	0 (0)		< 0.001	
Comorbidities										
Diabetes mellitus	33 (35.9)	39 (34.5)	0.956	0.028		19 (50.0)	16 (42.1)	0.645	0.165	0.020
Hypertension	19 (20.7)	25 (22.1)	0.933	0.036		7 (18.4)	8 (21.1)	> 0.999	0.065	0.166
Smoking	24 (26.1)	20 (17.7)	0.199	0.204		9 (23.7)	10 (26.3)	> 0.999	0.060	0.021
His_PONV	0 (0.0)	2 (1.8)	0.570	0.190		0 (0)	0 (0)	N/A	< 0.001	0.141
His_MS	2 (2.2)	3 (2.7)	1.000	0.031		0 (0)	0 (0)	N/A	< 0.001	0.043
*Intraoperative variables*										
Types of surgery										
LSG	21 (22.8)	88 (77.9)	< 0.001^*d*^	1.419		16 (42.1)	17 (44.7)	0.996	0.063	0.112
LSG-JJB	65 (70.7)	18 (15.9)				18 (47.4)	17 (44.7)		0.058	
LSG-DJB	5 (5.4)	3 (2.7)				3 (7.9)	3 (7.9)		< 0.001	
OAGB	1 (1.1)	4 (3.5)				1 (2.6)	1 (2.6)		< 0.001	
Dur_anesthesia (h)	1.8 ± 0.4	1.4 ± 0.3	< 0.001^*d*^	0.972		1.6 ± 0.4	1.6 ± 0.4	0.684	0.095	0.051
Dur_operation (h)	1.5 ± 0.4	1.2 ± 0.3	< 0.001^*d*^	0.958		1.4 ± 0.4	1.3 ± 0.4	0.849	0.047	0.050
IOC (mg)	60.5 ± 8.7	57.5 ± 7.2	0.008^*d*^	0.375		59.4 ± 8.2	58.1 ± 7.2	0.488	0.143	0.035
PHC	59 (64.1)	71 (62.8)	0.963	0.027		23 (60.5)	23 (60.5)	> 0.999	< 0.001	0.060
Total fluid input (mL)	1493.5 ± 379.9	1327.0 ± 330.3	0.001^*d*^	0.468		1379.0 ± 359.6	1400.0 ± 350.3	0.797	0.055	0.076
*Postoperative variables*										
Rescue opioids	59 (64.1)	61 (54.0)	0.185	0.207		20 (52.6)	22 (57.9)	0.818	0.110	0.067

Categorical data are presented as *n* (%), and continuous data are presented as mean ± standard deviation

*Abbreviations: ASA* American Society of Anesthesiologists, *ASD* Absolute standardised mean difference, *BMI* body mass index, *Dur_anesthesia* duration of the anesthesia, *Dur_operation* duration of the operation, *His_MS* history of motion sickness, *His_PONV* history of PONV, *IOC* intraoperative opioids consumption (as intravenous morphine equivalent), *LSG* laparoscopic sleeve gastrectomy, *LSG-DJB* laparoscopic sleeve gastrectomy plus duodenojejunal bypass, *LSG-JJB* laparoscopic sleeve gastrectomy plus jejunojejunal bypass, *N/A* not applicable, *OAGB* one anastomosis gastric bypass, *PHC* penehyclidine hydrochloride administration, *PONV* postoperative nausea and vomiting, *PSM* propensity score matching

^*a*^ Propensity score matching (PSM) was perform 1:1 using nearest neighbor method within a caliper width of 0.1 of the standard deviation without replacement

^*b*^ Inverse probability of treatment weighting (IPTW) method was used to control for observed confounding between the compared groups

^*c*^ Absolute standardized mean difference (ASD) was calculated to compare the two matched groups after matching. Variables with ASD > 0.2 were considered to be imbalanced

^*d*^ Statistically significant (*P* < 0.05)

As shown in Supplementary Tables 2, PONV occurred in 89 (43.4%) patients within the first 24 h and 27 (13.2%) within 24–48 h after LBS in the unmatched cohort. Patients received sugammadex were associated with significantly lower incidence of PONV (14.1% vs. 67.3%, OR 0.08, 95% CI 0.04–0.16, *P* < 0.001), and POV (8.7% vs. 54.0%, OR 0.08, 95% CI 0.04–0.18, *P* < 0.001) within postoperative 48 h. Reduced need for rescue antiemetic therapy (4.3% vs. 33.6%, OR 0.09, 95% CI 0.03–0.26, *P* < 0.001) and increased water intake (1882.4 ± 485.5 mL vs.1436.5 ± 526.4 mL, MD 445.8, 95% CI 305.1–586.6, *P* < 0.001) were observed in the sugammadex group (Table 4). Specifically, Patients received sugammadex were associated with lower incidence of PON only within 24–48 h after LBS (2.2% vs. 13.3%, OR 0.15, 95% CI 0.03–0.65, *P* = 0.004) and POV only within the first 24 h (8.7% vs. 54.0%, OR 0.08, 95% CI 0.04–0.18, *P* < 0.001) (Supplementary Table 2).

**Table 4:** Comparison of primary and secondary outcomes within 48 h postoperatively

Outcomes	Unmatched					After PSM					After IPTW	
	Sugammadex (*n* = 92)	Neostigmine (*n* = 113)	OR/MD^*a*^ (95% CI)	*P* value		Sugammadex (*n* = 38)	Neostigmine (*n* = 38)	OR/MD^*a*^ (95% CI)	*P* value		OR/MD^*a*^ (95% CI)	*P* value
PONV	13 (14.1)	76 (67.3)	0.08 (0.04–0.16)	< 0.001^*b*^		5 (13.2)	26 (68.4)	0.07 (0.02–0.22)	< 0.001^*b*^		0.54 (0.48–0.61)	< 0.001^*b*^
PON	5 (5.4)	15 (13.3)	0.38 (0.13–1.08)	0.060		4 (10.5)	7 (18.4)	0.52 (0.14–1.90)	0.328		0.77 (0.67–0.88)	< 0.001^*b*^
POV	8 (8.7)	61 (54.0)	0.08 (0.04–0.18)	< 0.001^*b*^		1 (2.6)	19 (50.0)	0.03 (0.01–0.22)	< 0.001^*b*^		0.60 (0.53–0.68)	< 0.001^*b*^
Rescue antiemetic therapy	4 (4.3)	38 (33.6)	0.09 (0.03–0.26)	< 0.001^*b*^		1 (2.6)	16 (42.1)	0.04 (0.01–0.30)	< 0.001^*b*^		0.70 (0.62–0.80)	< 0.001^*b*^
Water intake	1882.4 ± 485.5	1436.5 ± 526.4	445.8 (305.1–586.6)	< 0.001^*b*^		1932.9 ± 407.2	1510.5 ± 474.2	422.4 (220.3–624.4)	0.001^*b*^		419.5 (214.2–613.6)	< 0.001^*b*^

Data are presented as mean ± standard deviation (SD), median (IQR) or *n* (%) as appropriate

*Abbreviations: CI* confidence interval, *MD* mean difference, *N/A* not applicable, *OR* odds ratio, *PONV* postoperative nausea and vomiting, *PON* postoperative nausea, *POV* postoperative vomiting

^*a*^ Effect size: OR for PONV, PON, POV and rescue antiemetic therapy, and MD for water intake

^*b*^ Statistically significant (*P* < 0.05)

**Fig. 2:**
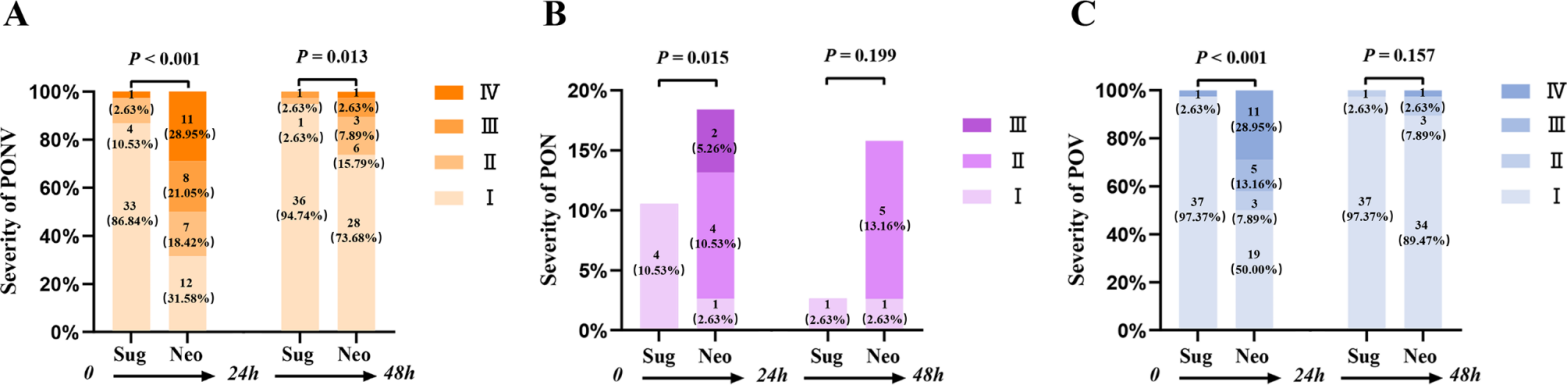
Stacked bar charts showing the severity of PONV (**A**), PON (**B**), and POV (**C**) within 48 h postoperatively between the sugammadex and neostigmine groups. *Sug* the sugammadex group, *Neo* the neostigmine group, *PONV* postoperative nausea and vomiting, *PON* postoperative nausea, *POV* postoperative vomiting

Subsequently, 38 patients were matched in each group after 1:1 PSM analysis. The logistic regression model for the matching was shown in Supplementary Table 3. After PSM and IPTW, balance between the two groups was achieved for all variables, with ASD < 0.2 (Table 3). Table 4 shows the primary and secondary outcomes regarding the risk of PONV, PON and POV between the two groups after PSM and IPTW. In the matched cohort, PONV occurred in 31 (40.8%) patients within the 48 h after LBS, including 5 (13.2%) in the sugammadex group and 26 (68.4%) in the neostigmine group. After IPTW adjustment, sugammadex use was associated with lower incidence of PONV (OR 0.54, 95% CI 0.48–0.61, *P* < 0.001) within postoperative 48 h. The incidence of POV (2.6% vs. 50.0%, OR 0.03, 95% CI 0.01–0.22, *P <* 0.001), but not PON, was significantly lower in the sugammadex group than those in the neostigmine group after PSM. However, the incidence of POV (OR 0.60, 95% CI 0.53–0.68, *P* < 0.001) and PON (OR 0.77, 95% CI 0.67–0.88, *P* < 0.001) within the postoperative 48 h were both lower in the sugammadex group after IPTW. In the subgroup analysis of the matched cohort, the occurrence of PONV (OR 0.16, 95% CI 0.03–0.77, *P* = 0.012) within 24–48 h after LBS, but neither PON nor POV, was less frequent in the sugammadex group than in the neostigmine group (Supplementary Table 4). The severity of PONV, PON and POV within the first 24 h were all significantly lower in the sugammadex group (*P <* 0.001; *P* = 0.015; *P <* 0.001, respectively) (Fig. 2). However, there was no significant association between sugammadex use and the severity of POV within 24–48 h after LBS after IPTW (Table 5).

**Table 5:** Comparison of primary and secondary outcomes within 48 h postoperatively after inverse probability of treatment weighting

Outcomes	0–24 h after surgery			24–48 h after surgery	
	OR/MD^*a*^ (95% CI)	*P* value		OR/MD^*a*^ (95% CI)	*P* value
PONV	0.53 (0.48–0.60)	< 0.001^*b*^		0.82 (0.74–0.91)	< 0.001^*b*^
Severity of PONV	0.25 (0.19–0.34)	< 0.001^*b*^		0.72 (0.58–0.90)	0.004^*b*^
PON	0.77 (0.67–0.88)	< 0.001^*b*^		0.89 (0.83–0.96)	0.004^*b*^
Severity of PON	0.58 (0.43–0.77)	< 0.001^*b*^		0.89 (0.83–0.97)	0.005^*b*^
POV	0.60 (0.53–0.68)	< 0.001^*b*^		0.91 (0.84–0.99)	0.022^*b*^
Severity of POV	0.30 (0.22–0.41)	< 0.001^*b*^		0.84 (0.69–1.03)	0.087
Rescue antiemetic therapy	0.70 (0.62–0.80)	< 0.001^*b*^		0.94 (0.88–1.01)	0.087
Water intake	170.5 (108.5–287.2)	< 0.001^*b*^		249.0 (115.2–420.4)	< 0.001^*b*^

*Abbreviations: CI* confidence interval, *MD* mean difference, *N/A* not applicable, *OR* odds ratio, *PONV* postoperative nausea and vomiting, *PON* postoperative nausea, *POV* postoperative vomiting

^*a*^ Effect size: OR for PONV, PON, POV and rescue antiemetic therapy, and MD for water intake

^*b*^ Statistically significant (*P* < 0.05)

The proportion of patients receiving postoperative rescue antiemetic therapy was lower in the sugammadex group than in the neostigmine group within the 48 h after LBS (2.6% vs. 42.1%; OR 0.04, 95% CI 0.01–0.30, *P* < 0.001) (Table 4). After IPTW, there was no significant association between sugammadex use and need for rescue antiemetic therapy within 24–48 h after LBS (Table 5). During the two periods (0–24 h and 24–48 h after LBS), water intake significantly increased in the sugammadex group (551.3 ± 149.5 mL vs. 404.0 ± 137.2 mL, MD 147.4, 95% CI 81.8–213.0, *P* < 0.001; 1381.6 ± 304.8 mL vs. 1106.6 ± 376.2 mL, MD 275.0, 95% CI 118.5–431.5, *P* = 0.001, respectively) (Supplementary Table 4).

**Fig. 3:**
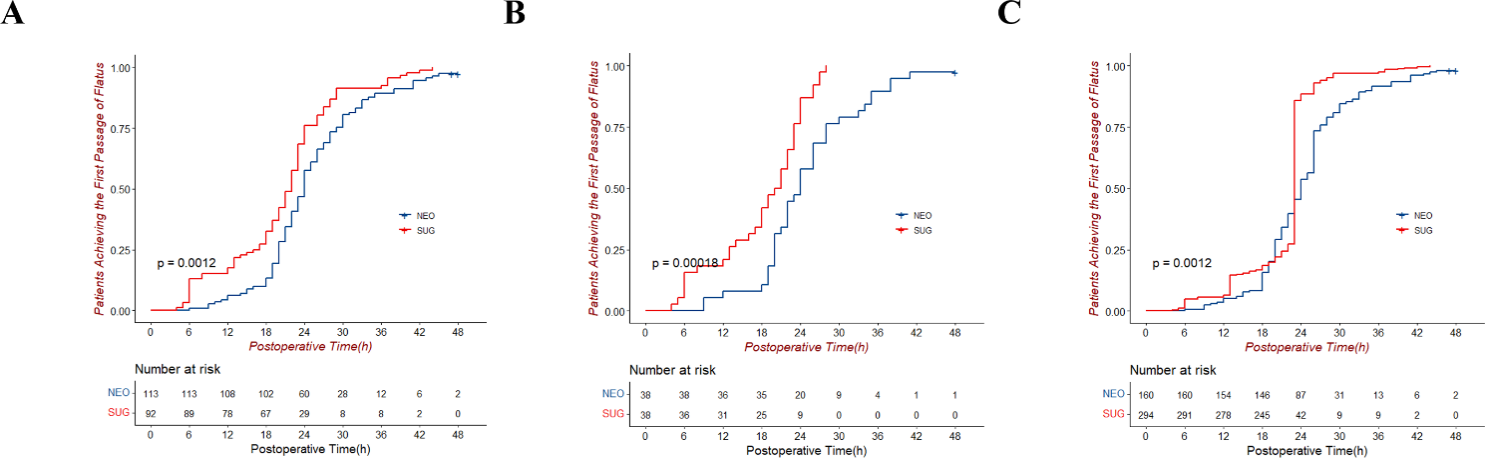
Kaplan-Meier curves for time to achieve the first passage of flatus postoperatively between the sugammadex and neostigmine groups. Unmatched cohort (**A**), cohort after propensity score matching (**B**), and cohort after inverse probability of treatment weighting (**C**)

With regard to the median onset time to the first passage of flatus, the patients in the sugammadex group had shorter time to first flatus after PSM and IPTW (*P* < 0.05), as shown by the Kaplan–Meier curves in Fig. 3.

## Discussion

A cohort study involving 74 patients undergoing LBS indicated that 59% of the patients experienced PONV despite being treated with supra-optimal PONV prophylaxis [2]. In the present study, although all patients with obesity received dexamethasone and palonosetron, the incidence of PONV in patients treated with neostigmine was as high as 67.3%. This result is consistent with those of previously published studies. Sugammadex use was an independent protective factor of PONV. Sugammadex dramatically reduced both the incidence and severity of PONV during postoperative hospital stay. In addition, it was associated with increased postoperative water intake and earlier first passage of flatus.

Bariatric surgery is a potential risk factor for PONV [13]. Gut vagal afferent fibers innervate the gastrointestinal tract and constitute a specific neural pathway to the nucleus of the solitary tract in the hindbrain that triggers vomiting [14]. Considering that surgical manipulation affects the gastric vagal nerve, the stimulation of these fibers may be responsible for the high incidence of PONV after LBS, especially in LSG, which would explain why LSG is more likely to potentiate PONV [5]. Moreover, CO_2_ pneumoperitoneum may play a role in the pathogenesis of PONV [15]. CO_2_ pneumoperitoneum contributes to increased intra-abdominal pressure and decreased intestinal blood flow, especially in obese patients. Aseptic inflammation caused by ischemia and hypoxia plays an important role in PONV by inducing the release of various transmitters [16]. The intestine is probably the most sensitive internal organ for ischemia. Therefore, short periods of ischemia can induce the release of neurotransmitters such as serotonin, which can stimulate the emetic chemoreceptor trigger zone [17, 18]. Given the high levels of serotonin in the gut, exposure of the gut to surgical procedures and anesthesia might increase the excitability of gut-vagus-brain reflex [19]. Furthermore, sugammadex is known to rapidly and reliably reverse NMB, which is beneficial for respiratory muscle strength recovery and CO_2_ exhalation. In addition, sugammadex might decrease hypoxic episodes after thoracic surgery compared with neostigmine [20]. Thus, patients receiving sugammadex might experience less postoperative residual CO_2_ and intestinal ischemia. In addition, PONV is associated with higher gastric intraluminal pressure and lower distensibility after bariatric surgery [21].

Two points need to be further explained. First, neostigmine is a cholinesterase inhibitor commonly used as an antagonist for NMBDs-induced neuromuscular paralysis. Theoretically, neostigmine is thought to increase the risk of PONV, possibly by provoking gastric spasms, lowering barrier pressure, and increasing afferent input to central vomiting centers [22]. However, neostigmine with a dose ≥ 2.5 mg increases the risk of PONV [23]. In addition, evidence-based analyses have suggested that there is insufficient evidence to conclude that routine dosage of neostigmine increases the risk of PONV [24, 25]. We avoided the administration of neostigmine > 2 mg (approximately equal to 10–25 mcg/kg lean body weight) to avoid increasing the incidence of PONV in our study. Second, the differences in the selection of NMBDs may call into question whether the use of these medications is associated with PONV in this setting. Rocuronium and cis-atracurium are both intermediate-acting non-depolarizing NMBDs, which means that the effects of the two drugs are comparable. Both groups strictly followed the same indications for extubation and discharge from the PACU. Considering that our primary outcome is the effect of sugammadex on PONV during postoperative stay in the ward, it seems unlikely that the choice of NMBDs would influence PONV long after administration.

Postoperative water intake and time to first flatus are two important indicators for evaluating recovery of gastrointestinal function. Early oral hydration following laparoscopic cholecystectomy is associated with lower incidence of PONV in the ward [26]. Neostigmine plus atropine was not correlated with postoperative bowel function recovery [27]. Patients receiving sugammadex experienced less PONV and were thus, prone to drinking water. Early oral hydration and increased water intake would help recover postoperative gastrointestinal tract function and accelerate gastric emptying, which is beneficial for lowering PONV. Compared with pyridostigmine-glycopyrrolate mixture, sugammadex shortened the time to first flatus after laparoscopic cholecystectomy [28]. Sugammadex binding gastric emptying-related steroid hormones, such as progesterone and estradiol, may potentiate the shortened time to first flatus [27, 29]. Those results are in accordance with our findings.

This present study had some limitations. First, only patients with a BMI ≥ 35 kg/m^2^ undergoing elective LBS are recommended to receive sugammadex for NMB reversal in our center. Thus, we enrolled only this specific population, leading to a relatively small sample size, which may have inherent limitations in the generalizability of our results. Second, although we have used several models to improve the robustness of statistical methods, stepwise variable selection has been criticized for being overly simplistic and prone to errors, which can lead to overfitting and might produce unstable and unreliable results particularly when there are many potential predictor variables. We found that the sugammadex use was still one of the independent protective factors of PONV in the least absolute shrinkage and selection operator regression. Therefore, we still selected univariate regression analysis and stepwise forward regression model to determine the influence of each variable on PONV in the present study. In the furture, there is a need for improved statistical methods for variable selection to avoid overfitting and stabilize the model. Third, it is not necessary to limit the ratio of PSM as 1:1. A more flexible 1:N matching could also be considered, which may result in less reduction of the patients due to the matching process. Fourth, for proper interpretation of the effects of sugammadex on PONV, similar NMB should have been used. However, a long time difference between NMB administration and PONV is no valuable argument for this kind of randomization. Finally, we did not collect data regarding the effects of sugammadex and neostigmine on late PONV because the majority of the patients in our center were discharged 48 h after LBS.

## Conclusions

In conclusion, compared with neostigmine, sugammadex can reduce the incidence and severity of PONV, increase postoperative water intake, and shorten the time to first flatus in bariatric patients during postoperative inpatient hospitalization, which may play a pivotal role in enhanced recovery.

## Electronic supplementary material

Below is the link to the electronic supplementary material.

**Supplementary Table 1** Risk factors of PONV using stepwise backward regression model (*n* = 205)

**Supplementary Table 2** Comparison of postoperative outcomes within 48 h in unmatched cohort

**Supplementary Table 3** The logistic regression model for the matching

**Supplementary Table 4** Comparison of primary and secondary outcomes within 48 h postoperatively between the matched groups

## Data Availability

The datasets generated and/or analyzed during the current study are not publicly available due to potential patient privacy compromise but are available from the corresponding author on reasonable request.

## Abbreviations

ASA: American Society of Anesthesiologists
ASD: Absolute standardized mean difference
BMI: Body mass index
CI: Confidence interval
Dur_anesthesia: Duration of the anesthesia
Dur_operation: Duration of the operation
His_MS: History of motion sickness
His_PONV: History of postoperative nausea and vomiting
IOC: Intraoperative opioids consumption
IPTW: Inverse probability of treatment weighting
LBS: Laparoscopic bariatric surgery
LSG: Laparoscopic sleeve gastrectomy
LSG-DJB: Laparoscopic sleeve gastrectomy plus duodenojejunal bypass
LSG-JJB: laparoscopic sleeve gastrectomy plus jejunojejunal bypass
MD: Mean difference
N/A: Not applicable
NMB: Neuromuscular blockade
NMBDs: Neuromuscular blocking drugs
OAGB: One anastomosis gastric bypass
OR: Odds ratio
PACU: Post anesthesia care unit
PHC: Penehyclidine hydrochloride
PHCBS: A trial exploring the effect of penehyclidine hydrochloride on postoperative nausea and vomiting in bariatric surgery
PON: Postoperative nausea
PONV: Postoperative nausea and vomiting
POV: Postoperative vomiting
PSM: Propensity score matching.
