# Supplementary material for: Use of sugammadex is associated with reduced incidence and severity of postoperative nausea and vomiting in adult patients with obesity undergoing laparoscopic bariatric surgery: a post-hoc analysis
Source: BMC Anesthesiol. 2023 May 15;23:163. doi: 10.1186/s12871-023-02123-y (PMC10184386; doi:10.1186/s12871-023-02123-y)
Supplement: Supplementary file 1 — Supplementary Table 1 Risk factors of PONV using stepwise backward regression model (n = 205) [file 12871_2023_2123_MOESM1_ESM.docx]

**Supplementary Table 1** Risk factors of PONV using stepwise backward regression model (*n* = 205)

| Variables | Univariate | |  | Multivariable*^a^* | |
| --- | --- | --- | --- | --- | --- |
|  | OR (95% CI) | *P* value |  | OR (95% CI) | *P* value |
| Age | 0.98 (0.94–1.01) | 0.188 |  |  |  |
| Female sex | 2.70 (1.43–5.08) | 0.002*^b^* |  | 3.45 (1.44–8.27) | 0.005*^b^* |
| BMI | 0.90 (0.85–0.96) | 0.001*^b^* |  |  |  |
| ASA classification |  |  |  |  |  |
| Ⅱ | 1 | Ref |  |  |  |
| Ⅲ | 5.55 (2.92–10.53) | < 0.001*^b^* |  |  |  |
| Apfel risk score |  |  |  |  |  |
| 0 | 1 | Ref |  |  |  |
| 1 | 1.50 (0.28–8.14) | 0.638 |  |  |  |
| 2 | 3.42 (0.70–16.76) | 0.129 |  |  |  |
| 3 | 3.21 (0.64–16.18) | 0.157 |  |  |  |
| 4 | N/A | > 0.999 |  |  |  |
| Diabetes mellitus | 0.37 (0.20–0.70) | 0.002*^b^* |  | 0.18 (0.08–0.43) | < 0.001*^b^* |
| Hypertension | 0.95 (0.48–1.89) | 0.893 |  |  |  |
| Smoking | 0.37 (0.17–0.81) | 0.012*^b^* |  |  |  |
| His_PONV | N/A | > 0.999 |  |  |  |
| His_MS | N/A | > 0.999 |  |  |  |
| Type of surgery |  |  |  |  |  |
| LSG | 1 | Ref |  |  |  |
| LSG­-JJB | 0.29 (0.15–0.54) | < 0.001*^b^* |  |  |  |
| LSG-DJB | N/A | > 0.999 |  |  |  |
| OAGB | 0.21 (0.02–1.96) | 0.171 |  |  |  |
| Dur_anesthesia | 0.21 (0.09–0.49) | < 0.001*^b^* |  |  |  |
| Dur_operation | 0.23 (0.10–0.54) | 0.001*^b^* |  |  |  |
| IOC | 0.94 (0.90–0.98) | 0.002*^b^* |  | 0.94 (0.89–0.99) | 0.015*^b^* |
| PHC | 0.68 (0.38–1.22) | 0.196 |  |  |  |
| Sugammadex | 0.05 (0.02–0.12) | < 0.001*^b^* |  | 0.03 (0.01–0.09) | < 0.001*^b^* |
| Total fluid input | 0.99 (0.98–1.00) | 0.003*^b^* |  |  |  |
| Rescue opioids | 0.82 (0.46–1.44) | 0.484 |  |  |  |

*Abbreviations: ASA* American Society of Anesthesiologists, *BMI* body mass index, *CI* confidence interval, *Dur_anesthesia* duration of the anesthesia, *Dur_operation* duration of the operation, *His_MS* history of motion sickness, *His_PONV* history of PONV, *IOC* intraoperative opioids consumption (as intravenous morphine equivalent), *LSG* laparoscopic sleeve gastrectomy, *LSG-DJB* laparoscopic sleeve gastrectomy plus duodenojejunal bypass, *LSG-JJB* laparoscopic sleeve gastrectomy plus jejunojejunal bypass, *N/A* not applicable, *OAGB* one anastomosis gastric bypass, *OR* odds ratio, *PHC* penehyclidine hydrochloride administration, *PONV* postoperative nausea and vomiting.

*^a^* Overall *P* value is less than 0.001 in Omnibus test of model coefficient.

*^b^* Statistically significant (*P* < 0.05).
