# Supplementary material for: Use of sugammadex is associated with reduced incidence and severity of postoperative nausea and vomiting in adult patients with obesity undergoing laparoscopic bariatric surgery: a post-hoc analysis
Source: BMC Anesthesiol. 2023 May 15;23:163. doi: 10.1186/s12871-023-02123-y (PMC10184386; doi:10.1186/s12871-023-02123-y)
Supplement: Supplementary file 2 — Supplementary Table 2 Comparison of postoperative outcomes within 48 h in unmatched cohort [file 12871_2023_2123_MOESM2_ESM.docx]

**Supplementary Table 2** Comparison of postoperative outcomes within 48 h in unmatched cohort

| Outcomes | 0–24 h after surgery | | | |  | 24–48 h after surgery | | | |
| --- | --- | --- | --- | --- | --- | --- | --- | --- | --- |
|  | Sugammadex  (*n* = 92) | Neostigmine  (*n* = 113) | OR/MD*^a^*  (95% CI) | *P* value |  | Sugammadex  (*n* = 92) | Neostigmine  (*n* = 113) | OR/MD*^a^*  (95% CI) | *P* value |
| PONV | 13 (14.1) | 76 (67.3) | 0.08  (0.04–0.16) | < 0.001*^b^* |  | 3 (3.3) | 24 (21.2) | 0.13  (0.04–0.43) | < 0.001*^b^* |
| PON | 5 (5.4) | 15 (13.3) | 0.38  (0.13–1.08) | 0.060 |  | 2 (2.2) | 15 (13.3) | 0.15  (0.03–0.65) | 0.004*^b^* |
| POV | 8 (8.7) | 61 (54.0) | 0.08  (0.04–0.18) | < 0.001*^b^* |  | 1 (1.1) | 9 (8.0) | 0.13  (0.02–1.02) | 0.051 |
| Rescue antiemetic therapy | 4 (4.3) | 38 (33.6) | 0.09  (0.03–0.26) | < 0.001*^b^* |  | 0 (0) | 5 (4.4) | N/A | 0.112 |
| Water intake | 543.8 ± 173.7 | 409.6 ± 158.1 | 134.2  (88.5–180.0) | < 0.001*^b^* |  | 1338.6 ± 353.8 | 1027.0 ± 405.8 | 311.6  (205.5–417.7) | < 0.001*^b^* |

Categorical data are presented as *n* (%), and continuous data are presented as mean ± standard deviation.

*Abbreviations: CI* confidence interval, *MD* mean difference, *N/A* not applicable, *OR* odds ratio, *PONV* postoperative nausea and vomiting, *PON* postoperative nausea, *POV* postoperative vomiting.

*^a^* Effect size: OR for PONV, PON, POV and rescue antiemetic therapy, and MD for water intake.

*^b^* Statistically significant (*P* < 0.05).
