# Supplementary material for: Use of sugammadex is associated with reduced incidence and severity of postoperative nausea and vomiting in adult patients with obesity undergoing laparoscopic bariatric surgery: a post-hoc analysis
Source: BMC Anesthesiol. 2023 May 15;23:163. doi: 10.1186/s12871-023-02123-y (PMC10184386; doi:10.1186/s12871-023-02123-y)
Supplement: Supplementary file 3 — Supplementary Table 3 The logistic regression model for the matching [file 12871_2023_2123_MOESM3_ESM.docx]

**Supplementary Table 3** The logistic regression model for the matching

| Variables | Coefficients | OR (95% CI) | *P* value |
| --- | --- | --- | --- |
| Age | 0.01 | 1.01 (0.96–1.07) | 0.742 |
| Female sex | 0.32 | 1.38 (0.37–5.23) | 0.633 |
| BMI | 0.08 | 1.08 (0.98–1.19) | 0.107 |
| ASA classification |  |  |  |
| Ⅱ | Ref | | |
| Ⅲ | 1.88 | 6.57 (1.81–23.83) | 0.004 |
| Apfel risk score |  |  |  |
| 0 | Ref | | |
| 1 | 0.95 | 2.58 (0.46–14.55) | 0.284 |
| 2 | 0.87 | 2.39 (0.25–22.73) | 0.447 |
| 3 | 1.07 | 2.92 (0.24–36.41) | 0.405 |
| 4 | 1.47 | N/A | > 0.999 |
| Diabetes mellitus | -0.77 | 0.46 (0.18–1.20) | 0.113 |
| Hypertension | 0.13 | 1.13 (0.40–3.23) | 0.814 |
| Smoking | 0.57 | 1.77 (0.41–7.61) | 0.443 |
| His_PONV | -19.50 | N/A | > 0.999 |
| His_MS | 0.42 | N/A | > 0.999 |
| Type of surgery |  |  |  |
| LSG | Ref | | |
| LSG­-JJB | 1.27 | 3.56 (1.24–10.20) | 0.018 |
| LSG-DJB | 1.29 | 3.62 (0.52–25.17) | 0.194 |
| OAGB | -0.75 | 0.47 (0.03–8.37) | 0.609 |
| Dur_anesthesia | 2.01 | 7.49 (0.13–438.37) | 0.332 |
| Dur_operation | -1.08 | 0.34 (0.01–21.69) | 0.611 |
| IOC | 0.06 | 1.06 (1.00–1.12) | 0.038 |
| PHC | -0.27 | 0.76 (0.34–1.73) | 0.516 |
| Total fluid input | 0.001 | 1.00 (0.998–1.001) | 0.730 |

*Abbreviations: ASA* American Society of Anesthesiologists, *BMI* body mass index, *Dur_anesthesia* duration of the anesthesia, *Dur_operation* duration of the operation, *His_MS* history of motion sickness, *His_PONV* history of PONV, *IOC* intraoperative opioids consumption (as intravenous morphine equivalent), *LSG* laparoscopic sleeve gastrectomy, *LSG-DJB* laparoscopic sleeve gastrectomy plus duodenojejunal bypass, *LSG-JJB* laparoscopic sleeve gastrectomy plus jejunojejunal bypass, *N/A* not applicable, *OAGB* one anastomosis gastric bypass, *PHC* penehyclidine hydrochloride administration, *PONV* postoperative nausea and vomiting.
