# Supplementary material for: Use of sugammadex is associated with reduced incidence and severity of postoperative nausea and vomiting in adult patients with obesity undergoing laparoscopic bariatric surgery: a post-hoc analysis
Source: BMC Anesthesiol. 2023 May 15;23:163. doi: 10.1186/s12871-023-02123-y (PMC10184386; doi:10.1186/s12871-023-02123-y)
Supplement: Supplementary file 4 — Supplementary Table 4 Comparison of primary and secondary outcomes within 48 h postoperatively between the matched groups [file 12871_2023_2123_MOESM4_ESM.docx]

**Supplementary Table 4** Comparison of primary and secondary outcomes within 48 h postoperatively between the matched groups

| Outcomes | 0–24 h after surgery | | | |  | 24–48 h after surgery | | | |
| --- | --- | --- | --- | --- | --- | --- | --- | --- | --- |
|  | Sugammadex  (*n* = 38) | Neostigmine  (*n* = 38) | OR/MD*^a^*  (95% CI) | *P* value |  | Sugammadex  (*n* = 38) | Neostigmine  (*n* = 38) | OR/MD*^a^* (95% CI) | *P* value |
| PONV | 5 (13.2) | 26 (68.4) | 0.07  (0.02–0.22) | < 0.001*^b^* |  | 2 (5.3) | 10 (26.3) | 0.16  (0.03–0.77) | 0.012*^b^* |
| PON | 4 (10.5) | 7 (18.4) | 0.52  (0.14–1.9) | 0.328 |  | 1 (2.6) | 6 (15.8) | 0.14  (0.02–1.26) | 0.113 |
| POV | 1 (2.6) | 19 (50.0) | 0.03  (0.01–0.22) | < 0.001*^b^* |  | 1 (2.6) | 4 (10.5) | 0.23  (0.02–2.16) | 0.355 |
| Rescue antiemetic therapy | 1 (2.6) | 16 (42.1) | 0.04  (0.01–0.30) | < 0.001*^b^* |  | 0 (0) | 2 (5.3) | N/A | 0.474 |
| Water intake | 551.3 ± 149.5 | 404.0 ± 137.2 | 147.4  (81.8–213.0) | < 0.001*^b^* |  | 1381.6 ± 304.8 | 1106.6 ± 376.2 | 275.0  (118.5–431.5) | 0.001*^b^* |

Categorical data are presented as *n* (%), and continuous data are presented as mean ± standard deviation.

*Abbreviations: CI* confidence interval, *MD* mean difference, *N/A* not applicable, *OR* odds ratio, *PONV* postoperative nausea and vomiting, *PON* postoperative nausea, *POV* postoperative vomiting.

*^a^* Effect size: OR for PONV, PON, POV and rescue antiemetic therapy, and MD for water intake.

*^b^* Statistically significant (*P* < 0.05).
